# Cucumber Ribosomal Protein CsRPS21 Interacts With P22 Protein of Cucurbit Chlorotic Yellows Virus

**DOI:** 10.3389/fmicb.2021.654697

**Published:** 2021-04-29

**Authors:** Xue Yang, Ying Wei, Yajuan Shi, Xiaoyu Han, Siyu Chen, Lingling Yang, Honglian Li, Bingjian Sun, Yan Shi

**Affiliations:** College of Plant Protection, Henan Agricultural University, Zhengzhou, China

**Keywords:** cucurbit chlorotic yellows virus, P22, ribosomal protein, silencing suppressor, virus accumulation

## Abstract

Cucurbit chlorotic yellows virus (CCYV) is a cucurbit-infecting crinivirus. RNA silencing can be initiated as a plant defense against viruses. Viruses encode various RNA silencing suppressors to counteract antiviral silencing. P22 protein encoded by RNA1 of CCYV is a silencing suppressor, but its mechanism of action remains unclear. In this study, the cucumber ribosomal-like protein CsRPS21 was found to interact with P22 protein *in vitro* and *in vivo*. A conserved CsRPS21 domain was indispensable for its nuclear localization and interaction with P22. Transient expression of CsRPS21 in *Nicotiana benthamiana* leaves interfered with P22 accumulation and inhibited P22 silencing suppressor activity. CsRPS21 expression in *N. benthamiana* protoplasts inhibited CCYV accumulation. Increasing numbers of ribosomal proteins are being found to be involved in viral infections of plants. We identified a P22-interacting ribosomal protein, CsRPS21, and uncovered its role in early viral replication and silencing suppressor activity. Our study increases knowledge of the function of ribosomal proteins during viral infection.

## Introduction

The members of the genus *Crinivirus* cause significant yield and quality losses in many cucurbit species (Kreuze et al., 2005; Navas-Castillo et al., 2011). Like most members of the genus *Crinivirus*, cucurbit chlorotic yellows virus (CCYV) has a bipartite genome. The genomes of criniviruses vary among species in the RNA1 3′ region downstream from RNA-dependent polymerase coding region, where the number of open reading frames (ORFs) varies from zero to three (Kataya et al., 2009). CCYV RNA1 contains four ORFs: ORF1a, ORF1b, ORF2, and ORF3. ORF1a encodes viral methyltransferase and RNA helicase 1; ORF1b encodes an RNA-dependent RNA polymerase motif; and ORF2 and ORF3 encode the predicted proteins P6 and P22, respectively. As RNA1 3′ ORFs are quite variable among criniviruses, both P6 and P22 show no significant similarity to corresponding proteins of other criniviruses (Okuda et al., 2010). In addition to CCYV P22, P22 proteins of tomato chlorosis virus (ToCV) and sweet potato chlorotic stunt virus (SPCSV), P23 of lettuce chlorosis virus (LCV), and P25 of cucurbit yellow stunting disorder virus (CYSDV) have also been identified as RNA silencing suppressors (Kreuze et al., 2005; Canizares et al., 2008; Kataya et al., 2009; Kubota and Ng, 2016; Orfanidou et al., 2019; Salavert et al., 2020).

Ribosomal proteins (RPs) are named according to their association with the small or large ribosomal subunit (Kaltschmidt and Wittmann, 1970). In *Arabidopsis*, 81 different RPs encoded by 242 putatively functional RP genes have been identified (Hummel et al., 2015). Apart from RNA chaperone activity, some RPs regulate processes related to the cell cycle, apoptosis, development, oncogenesis, and rDNA transcription (Chen and Ioannou, 1999; Panic et al., 2007; Jeon et al., 2008; Lindstrom, 2009; Kim et al., 2014; Rajamaki et al., 2017). In plant viruses, P6 of cauliflower mosaic virus (CaMV) is found to interact with RPs L18, L24, and L13 (Bureau et al., 2004). These interactions may be involved in the translation reinitiation of polycistronic mRNAs (Bureau et al., 2004). RP L10 serves as the substrate for the kinase domain of nuclear shuttle protein interacting kinase, which is identified as a virulence target of the begomovirus nuclear shuttle protein and negatively affected tomato golden mosaic virus (TGMV) and tomato crinkle leaf yellows virus (TCrLYV) infection (Fontes et al., 2004; Rocha et al., 2008; Zorzatto et al., 2015). Many RP genes in *N. benthamiana* are upregulated at the mRNA level in response to infection by turnip mosaic virus (TuMV) and plum pox virus (PPV) (Dardick, 2007; Yang et al., 2009). In the case of tomato ringspot virus (ToRSV), the expression of plastid ribosomal genes is repressed (Dardick, 2007). RP S6 in *N. benthamiana* is involved in infections by various viruses and affects the accumulation of cucumber mosaic virus (CMV), TuMV, and potato virus A (PVA), but not turnip crinkle virus (TCV) or tobacco mosaic virus (TMV) (Rajamaki et al., 2017). Although increasing numbers of RPs are involved in viral infections of plants (Chen and Ioannou, 1999; Bureau et al., 2004; Panic et al., 2007; Jeon et al., 2008; Rocha et al., 2008; Rajamaki et al., 2017), the role of RPS21 during viral infection, if any, has not been characterized yet. In this study, we identified a cucumber ribosomal-like protein CsRPS21 that interacts with CCYV P22 protein *in vitro* and *in vivo*. A conserved domain of CsRPS21 was found to be indispensable for its nuclear localization and the interaction with P22; CsRPS21 interfered with the P22 accumulation to inhibit P22 silencing suppressor activity and viral accumulation.

## Materials and Methods

### Plant Materials and Agrobacteria Inoculation

The *N. benthamiana* seeds were provided by Dr. Yanhong Qin from Henan Academy of Agricultural Sciences, and the plants were grown in pots in a growth room under a 16 h light/8 h dark photoperiod at 25°C with 60% humidity. For agroinfiltration, agrobacteria GV3101 carrying relevant clones were suspended in infiltration buffer (10 mM MgCl_2_, 10 mM MES, and 200 μM acetosyringone, pH 5.6) at an OD_600_ of 1, kept at room temperature for 2–4 h, and infiltrated into *N. benthamiana* leaves using a 1 ml needleless syringe.

### Yeast Two Hybrid Screen and Interaction Assay

The cucumber cDNA library screening was performed according to the protocol handbook provided by Matchmaker Gold Yeast Two-Hybrid System (Clontech, CA, United States). The cucumber library was used to screen P22 interacting proteins. The cDNA library screen and interaction assay were performed as described previously (Cheng et al., 2008).

### Plasmid Construction

Supplementary Table 1 has the information regarding primers used in this study. All the constructs used were sequenced before use.

To construct vectors for yeast two-hybrid analysis, the full length of CCYV P22 (KU507601) was amplified and cloned into yeast vector pGBKT7 at the *Nde*I and *Bam*HI sites to generate the bait vector BDP22 using the primer pair BDP22F and BDP22R. The full-length coding sequence of CsRPS21 (XM_004145623) was amplified using the primer pair ADCsRPS21F/ADCsRPS21R, and subcloned into the vector pGADT7 at the *Eco*RI and *Xho*I sites to generate ADCsRPS21. We used the primer pairs ADCsRPS21_9__1_F/ADCsRPS21R, ADCsRPS21F/ADCsRPS21_14__5_R, ADCsRPS21_12__8_F/ADCsRPS 21R, ADCsRPS21F/ADCsRPS21_12__7_R, ADCsRPS21_9__1_F/ADCs RPS21_14__5_R, ADCsRPS21F/ADCsRPS21_9__0_R, and subcloned into the vector pGADT7 at the *Eco*RI and *Xho*I sites to generate ADRP1, ADRP2, ADRP3, ADRP4, ADRP5, and ADRP6.

For bimolecular fluorescence complementation (BiFC) analysis, the relevant vectors of P22, CsRPS21, and CsRPS21_1–1__45_ were constructed using gateway strategy. P22, CsRPS21, and CsRPS21_1–1__45_ were cloned into entry vector pDONR221 using primer pairs BPP22F/BPP22R, BPCsRPS21F/BPCsRPS21R, and BPCsRPS21F/BPCsRPS21_14__5_R to generate BPCsRPS21, BPCsRPS21_1–1__45_ and BPP22. The resultant clones were recombined into the binary expression vector pEarleyGate201-YN and pEarleyGate202-YC (Lu et al., 2010) by the LR reaction, constructing the gateway vector CsRPS21-cYFP, CsRPS21_1–1__45_-cYFP and P22-nYFP.

For nuclear localization assay, sequences corresponding to 91-145 aa, 1-127 aa and 128-183 aa of CsRPS21 were amplified and cloned into entry vector pDONR221 using primer pairs BPCsRPS21_9__1_F/BPCsRPS21_14__5_R, BPCsRPS21F/BPCsRPS21_12__7_R, and BPCsRPS21_12__8_F/BPCsRPS21R to generate BPCsRPS21_9__1–1__45_, BPCsRPS21_1–1__27_, and BPCsRPS21_12__8–1__83_. The resultant clones together with BPCsRPS21 were used to clone into the gateway vector pEarleyGate104 (Earley et al., 2006) to obtain the expression vector YFP-CsRPS21_9__1–1__45_, YFP-CsRPS21_1–1__27_, YFP-CsRPS21_12__8–1__83_, and YFP-CsRPS21. BPP22 was cloned into pEG104 and pEG100-RFP to acquire YFP-P22 and P22-RFP.

For interaction with other CCYV proteins, BD vectors of P4.9, RNA1P6, RNA2P6, HSP70h, P9, P26, CP, CPm, and P59 (KU507602) were constructed and stored in our lab (Wang et al., 2015).

For P22 silencing suppressor activity, pGDFLag-P22 was constructed by introducing P22 into pGDFlag (Goodin et al., 2002) vector. P22 were amplified using primer pair FLAGP22F/FLAGP22R. PCR product was digested by *Sal*I and *Bam*HI, and cloned into pGDFlag vector to generate pGDFlag-P22. pGDMyc-CsRPS21 was constructed by introducing CsRPS21 into pGDMyc (Goodin et al., 2002) vector. CsRPS21 were amplified using primer pair MycRPS21F/MycRPS21R, digested by *Pst*I and *Bam*HI, then cloned into pGDMyc vector to generate pGDMyc-CsRPS21.

### Confocal Laser Scanning Microscopy

For BiFC and co-localization assay, agrobacteria carrying the corresponding constructs were infiltrated into *N. benthamiana* leaves as described previously (Walter et al., 2004). The leaves were detached at 48 h post infiltration (hpi) for fluorescence detection. Fluorescence signals were visualized under an inverted spectral confocal laser scanning microscope (Cal Zeiss LSM 710). Fluorescence of YFP and RFP was excited at 514 and 561 nm. Fluorescence of chlorophy II was excited at 637 nm.

### Quantification of GFP Fluorescence Intensity

Images of GFP fluorescence from experimental and corresponding control plants were taken under the Nikon fluorescence microscope ECLIPSE Ti-S at 3 days post-infiltration (dpi). Thirty fluorescent spots were selected at random from two leaves, and the areas were measured using ImageJ2 software. Thirty independent images for each group were measured and values were analyzed via *t*-test. Three biological repeats were needed.

### Western Blotting

Agro-infiltrated leaves were harvested at 3 dpi for western blotting assay. Total protein was extracted from 0.2 g leaf tissues using the extraction buffer containing 20% glycerol, 20 mM Tris-HCl pH 7.5, 1 mM EDTA, 150 mM NaCl, 1 mM PMSF, 1 × Protease inhibitor cocktail (Sigma, China). Total protein was separated in SDS-polyacrylamide gel electrophoresis, followed by transfer to nitrocellulose membranes. The membranes were probed using polyclonal anti-GFP (Sigma, China), anti-Flag (Sigma, China), and anti-Myc (Abmart, China) antibody followed by an HRP-conjugated secondary antibody. The detection signals were developed using an ECL reagent as instructed.

### *N. benthamiana* Protoplasts Isolation and Transfection

Protoplasts were isolated from *N. benthamiana* seedlings and transfected using polyethylene glycol (PEG)-mediated method with modifications (Bak and Folimonova, 2015). Approximately 200 μl of protoplasts (2 × 10^5^) were gently mixed with 5 μg of CCYV RNA1 and RNA2 which were obtained from *in vitro* transcription and incubated at room temperature for 15 min. The protoplasts were gently washed in W5 solution and incubated in the dark at 25°C. The transfected protoplasts were harvested at 24 h post transfection and used for quantitative reverse transcription PCR (RT-qPCR) analysis. Three independent experiments were conducted and protoplasts from two tubes were pooled for RNA isolation followed by RT-qPCR analysis.

### Northern Blotting

Protoplasts, isolated from *N. benthamiana* seedlings and transfected with CCYV for 24 h, were sampled and pooled for total RNA extraction. RNA samples of 3 μg were used to detect CCYV RNA1 mRNA. Northern blotting analysis was conducted according to the manual of the Northern starter kit (Roche Diagnostics, Basel, Switzerland). RNA was labeled in an *in vitro* transcription reaction with CCYV RNA1 as a template using a labeling mixture. Supplementary Table 1 shows the probe primers used to detect the CCYV RNA1. The intensities of bands for RNA1 were normalized against the intensities of loading bands with the relative value of YFP control as 1.00. The quantitative calculation of digital images of blots was done using ImageJ2 software.

Transient co-expression of GFP and CsRPS21 or GUS (as a control protein) in GFP-transgenic *N. benthamiana* plants (16c) at 5 dpi, were sampled and pooled for total RNA extraction. RNA samples of 3 μg were used to detect GFP mRNA with GFP probe. The quantitative calculation of digital images of blots was done using ImageJ2 software.

### Quantitative Reverse Transcription PCR

Total RNA was extracted from harvested *N. benthamiana* protoplasts using Trizol reagent (Invitrogen, United States) and treated with RNase-free DNase I at 24 hpi. First strand cDNA was synthesized using 500 ng total RNA, an oligo d (T) primer, random primer, and M-MLV reverse transcriptase as instructed. Ten-fold diluted cDNA product was used for PCR on an Eppendorf Real-Time PCR system using an SYBR Green master mix (Takara, Japan). The *N. benthamiana* actin gene (AY179605) was used as the internal control. All the primers used for RT-qPCR are listed in Supplementary Table 1. The relative gene expression levels were calculated using the 2^−△△CT^ method (Livak and Schmittgen, 2001). Each treatment contains technical triplicates and three independent experiments to confirm the stable expression of genes of interest. *T*-test was performed on data using GraphPad Prism (Inc., San Diego, CA, United States). A two-sample unequal variance directional *t*-test was used to test the significance of the difference (^∗^*P* < 0.05; ^∗∗^*P* < 0.01).

## Results

### Identification of a P22-Interacting RPS21 Protein From Cucumber

**FIGURE 1:**
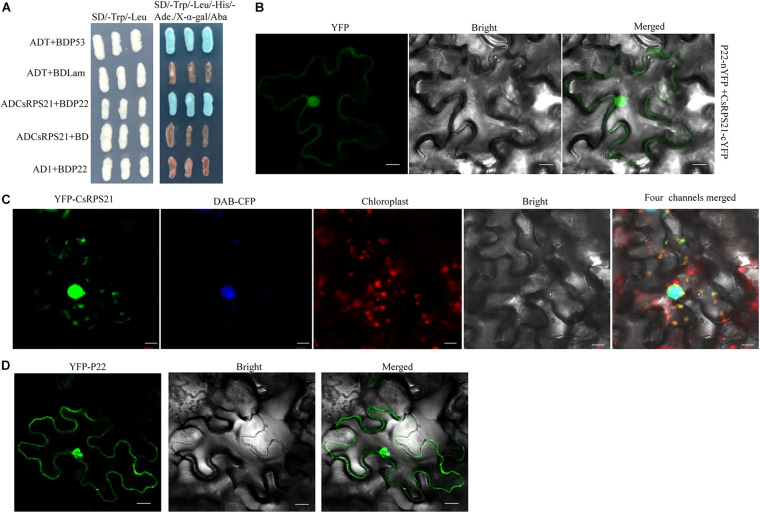
Identification of the interaction between P22 and CsRPS21. **(A)** Growth of Y2HGold yeast cells co-transformed with ADCsRPS21 and BDP22 on low-(SD/–Trp/–Leu) and high-(SD/–Trp/–Leu/–His/–Ade/Aba/X-α-gal) stringency selection media. ADT + BDP53 and ADT + BDLam served as positive and negative controls, respectively. **(B)** Visualization of the interaction between P22 and CsRPS21 in *N*. *benthamiana* epidermal cells using BiFC. P22 fused with the N-terminal portion of YFP (P22-nYFP) was transiently co-expressed with CsRPS21 fused with the C-terminal portion of YFP (CsRPS21-cYFP). Bar represents 10 μm. Photos were taken at 2 dpi using a Zeiss LSM710 laser scanning microscope. **(C)** Localization of CsRPS21 in *N*. *benthamiana* epidermal cells. YFP-tagged CsRPS21 (YFP-CsRPS21) was expressed *in planta*. Confocal images were taken at 2 dpi. Nuclear localization protein DAB-CFP was used as a nucleus indicator. Bar represents 10 μm. **(D)** Localization of P22 in *N*. *benthamiana* epidermal cells. YFP-tagged P22 (YFP-P22) was expressed *in planta*. Confocal images were taken at 2 dpi. Bar represents 10 μm.

To identify cucumber proteins that interact with CCYV P22 protein, we performed a yeast two-hybrid screen using a cucumber cDNA library (Chen et al., 2019). The coding sequence of P22 was placed in the pGBKT7 vector as bait. After screening, one clone that contained the entire ORF of a ribosomal-like protein was selected for further study (GenBank accession number XM_004145623). Using blastx, the ribosome-like protein identified here belonged to the ribosomal S21 superfamily, and showed 84.2% identity at the amino acid level with 30S RP S21 from *Cucurbita maxima*; hence, we designated it CsRPS21. Sequence analysis showed that RPS21 protein was conserved in cucurbits (Supplementary Figure 1). The CsRPS21 coding sequence was cloned into pGADT7 and the interaction with P22 was tested in the yeast strain Y2HGold using yeast co-transformation. The interaction was seen on SD/–Leu/–Trp/–His/–Ade/Aba/X-α-gal plates (Figure 1A). To confirm the interaction between P22 and CsRPS21 *in planta*, we used BiFC analysis to test the interaction. The coding sequences of CCYV P22 and CsRPS21 were cloned into pEG201-YN and pEG202-YC, respectively, to generate P22-nYFP and CsRPS21-cYFP for infiltration into leaves. Yellow fluorescent protein (YFP) fluorescence was detected in *N*. *benthamiana* leaves agroinfiltrated with P22-nYFP and CsRPS21-cYFP at 2 dpi. Fluorescence was observed mainly in the nucleus, with weak fluorescence in the cytoplasm (Figure 1B). No such interaction was found between P22-nYFP and cYFP or nYFP and CsRPS21-cYFP (Supplementary Figure 2). To determine if the interaction influenced the localization of CsRPS21 and P22, YFP-CsRPS21 and YFP-P22 were transiently expressed in *N*. *benthamiana* leaves. YFP-CsRPS21 fluorescence was found mainly in the nucleus and some chloroplasts (Figure 1C). YFP-P22 was observed in both the cytoplasm and nucleus (Figure 1D) suggesting that the interaction changed the localization of CsRPS21. Besides co-localization of YFP-CsRPS21 and P22-RFP in *N*. *benthamiana* leaves showed the co-localization of P22 and CsRPS21 in the nucleus (Supplementary Figure 3A).

### Interactions Between CsRPS21 and Other CCYV Proteins

**FIGURE 2:**
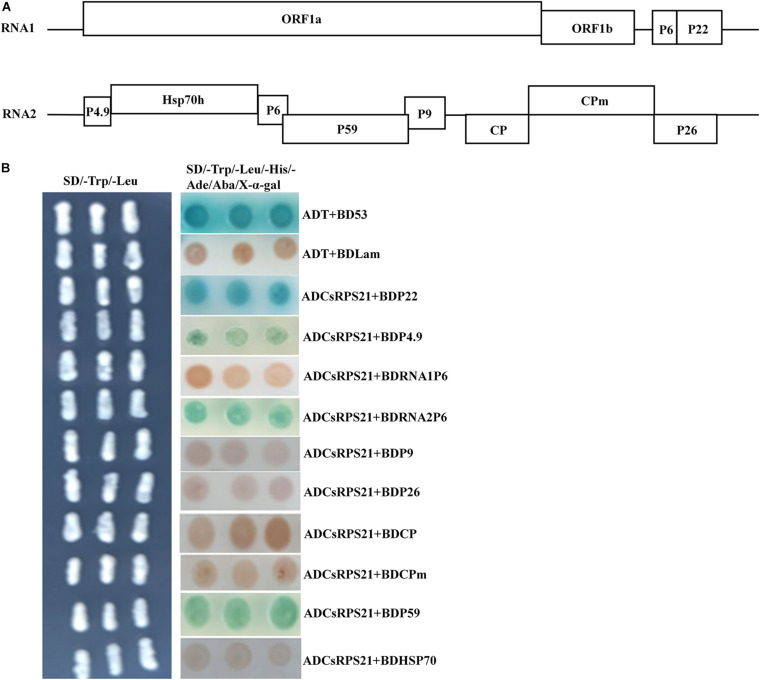
Identification of the interaction between CsRPS21 and CCYV proteins **(A,B)** ADCsRPS21 was co-transformed with CCYV proteins, as P4.9, P1-6, P2-6, P9, P26, CP, CPm, P59, and HSP70, into Y2HGold yeast cells and plated on a low-(SD/–Trp/–Leu) and high-(SD/–Trp/–Leu/–His/–Ade/Aba/X-α-gal) stringency selection media. ADT + BDP53 and ADT + BDLam served as positive and negative controls, respectively.

The interaction between CsRPS21 and other CCYV proteins was tested using yeast co-transformation methods. AD-CsRPS21 was co-transformed with BD vectors of P4.9, RNA1P6, RNA2P6, HSP70h, P9, P26, CP, CPm, and P59, and plated on SD/–Leu/–Trp/–His/–Ade/Aba/X-α-gal plates for selection. In addition to P22, interactions with CsRPS21 occurred with P4.9, RNA2P6, and P59 (Figure 2).

### The Conserved NLS Domain of CsRPS21 Is Indispensable for Nuclear Localization and P22-CsRPS21 Interaction

**FIGURE 3:**
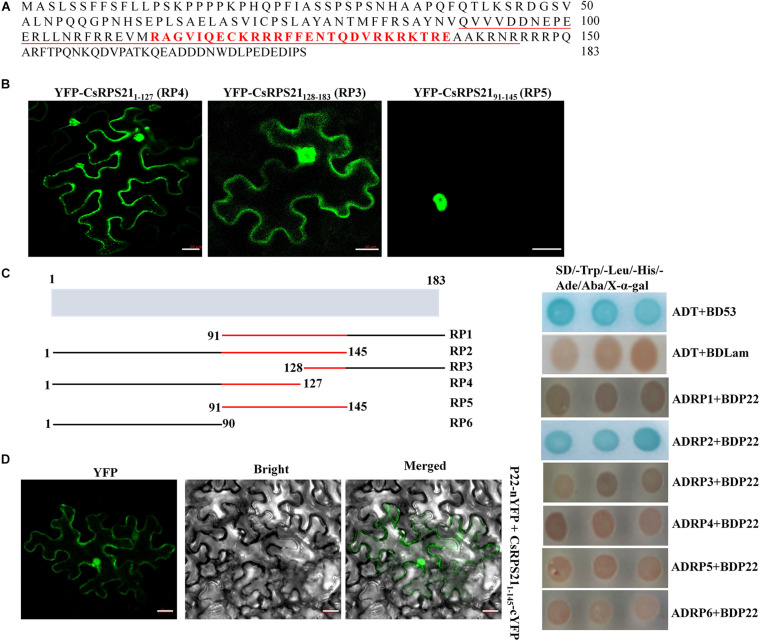
Nuclear localization signal determination and its involvement in the interaction. **(A)** The full amino acid sequence of CsRPS21. Amino acids constituting the nuclear localization signal predicted using cNLS mapper are shown in red. The conserved ribosomal protein S21 domain predicted using the SMART protein prediction tool is underlined. **(B)** Three YFP-tagged deletion mutants of CsRPS21 (YFP-CsRPS21_1–1__27_, YFP-CsRPS21_12__8–1__83_, and YFP-CsRPS21_9__1–1__45_) were transiently expressed in *N*. *benthamiana* leaves. Bar represents 20 μm. Photos were taken at 2 dpi using a Zeiss LSM710 laser scanning microscope. **(C)** Left panel: schematic representation of the CsRPS21 deletion mutants. Six CsPRS21 deletion mutants were used: RP1 (residues 91–183), RP2 (residues 1–145), RP3 (residues 128–183), RP4 (residues 1–127), RP5 (residues 91–145), and RP6 (residues 1–90). Right panel: Interaction between BDP22 and the CsRPS21 deletion mutants. Growth of Y2HGold yeast cells co-transformed with BDP22 and ADRP1, ADRP2, ADRP3, ADRP4, ADRP5, or ADRP6 on high-stringency selection medium (SD/–Leu/–Trp/–His/–Ade/Aba/X-α-gal). **(D)** Interaction between P22 and CsRPS21_1–1__45_ (RP2) in *N*. *benthamiana* epidermal cells using BiFC. P22 fused with the N-terminal portion of YFP (P22-nYFP) was transiently co-expressed with CsRPS21_1–1__45_ fused with the C-terminal portion of YFP (CsRPS21_1–1__45_-cYFP). Bar represents 20 μm. Photos were taken at 3 dpi using a Zeiss LSM710 laser scanning microscope.

Since the interaction was observed mainly in the nucleus, we identified the nuclear localization domain of CsRPS21 and tested its role in the interaction. Using the cNLS mapper program^1^, the nuclear localization signal was predicted to be located between amino acids 113 and 139 (Figure 3A). Using the SMART protein prediction tool, the conserved domain of RP S21 was identified at amino acids 91–145. Next, we constructed the CsRPS21 deletion mutants YFP-CsRPS21_9__1–1__45_ (RP5), YFP-CsRPS21_1–1__27_ (RP4), and YFP-CsRPS21_12__8–1__83_ (RP3) and observed fluorescence after agroinfiltration into leaves. The nuclear localization signal was found to be located between amino acids 91 and 145 (Figure 3B). We tested the necessity for the localization signal in the interaction of P22 and CsRPS21 using a series of CsRPS21 deletion constructs (Figure 3C, left). Yeast cotransformation results showed that AD-CsRPS21_1–1__45_ (RP2) interacted with BDP22, indicating that the N-terminal 145 amino acids containing the nuclear localization signal are necessary for the interaction (Figure 3C, right). Using BiFC analysis, YFP fluorescence was detected in leaves agroinfiltrated with P22-nYFP and CsRPS21_1–1__45_-cYFP (RP2) at 2 dpi (Figure 3D and Supplementary Figure 4).

### CsRPS21 Negatively Regulates P22 Silencing Suppressor Activity

**FIGURE 4:**
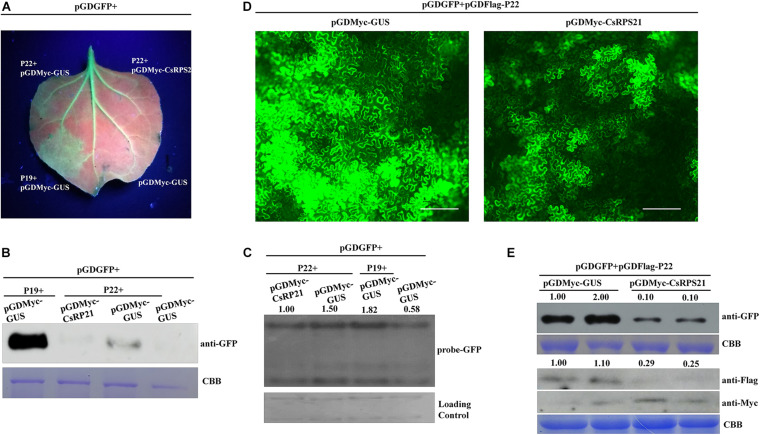
CsRPS21 negatively regulates P22 silencing suppressor activity **(A–C)** Silencing suppression ability of P22 was tested in 16c *N. benthamiana* plants, with transient co-expression of GFP and CsRPS21 or GUS (as a control protein). GFP fluorescence was revealed by UV illumination at 5 dpi with *Agrobacterium* constructs. Western blotting **(B)** and northern blotting **(C)** were used to detect the accumulation of GFP. **(D)** *N. benthamiana* leaves were infiltrated with a mixture of three *Agrobacterium* cultures carrying pGDGFP, pGDFlag-P22, pGDMyc-GUS, or pGDMyc-CsRPS21 and photographed at 3 dpi. Images of GFP fluorescence from agroinfiltrated leaves were taken under a Nikon ECLIPSE Ti-S fluorescence microscope. Bar represents 200 μm. **(E)** Western blotting analysis of protein extracted from the *N*. *benthamiana* leaves indicated above. GFP, CsRPS21 and P22 expression were confirmed using anti-GFP, anti-Myc and anti-Flag antibody. Coomassie Brilliant Blue (CBB) staining of the large subunit of Rubisco served as a loading control (Supplementary Figure 5).

Previously, P22 was identified as a weak silencing suppressor (Chen et al., 2019; Orfanidou et al., 2019). To determine whether CsRPS21 expression affects the P22 silencing suppressor activity, we tested the ability of P22 to suppress RNA silencing by ectopic expression of GFP together with CsRPS21 or GUS in *N*. *benthamiana* and GFP-transgenic *N. benthamiana* plants (16c) leaves. In these experiments, silencing of the GFP transgene was initiated by infiltration with an *Agrobacterium* culture carrying a second copy of the GFP gene. As shown in Figure 4A, at 5 dpi, the amount of GFP fluorescence was observable in leaf patches co-infiltrated with plasmids expressing GFP + P22 + pGDMyc-GUS and GFP + P19 + pGDMyc-GUS (as the positive control). These results demonstrated that P22 was able to suppress silencing of the GFP gene in these infiltrated patches. In contrast, GFP fluorescence was much lower in a patch co-infiltrated with GFP and P22 plus pGDMyc-CsRPS21, indicating that the pGDMyc-CsRPS21 could prevent P22 from suppressing silencing of the GFP gene. Western blotting and northern blotting were done to confirm that the levels of apparent GFP fluorescence correctly reflected the level of GFP protein and mRNA accumulation in the various patches (Figures 4B,C).

Using fluorescence microscopy, strong GFP fluorescence was observed in *N*. *benthamiana* leaves agroinfiltrated with pGDGFP and pGDFLag-P22 together with pGDMyc-GUS (as control treatment). When co-expression of pGDGFP + pGDFLag-P22 and pGDMyc-CsRPS21, the fluorescence was weaker than control indicating that CsRPS21 expression inhibits P22 silencing suppressor activity (Figure 4D). The fluorescence intensity per visual field of the GUS control was significantly higher than that with the pGDMyc-CsRPS21 treatment (Supplementary Figure 5). Western blotting showed that the expression of GFP was consistent with previous findings. We also detected the expression of pGDMyc-CsRPS21 and pGDFlag-P22. CsPRS21 inhibited the P22 expression (Figure 4E). Based on these results we speculate that CsRPS21 interfere with P22 expression to attenuate the RNA silencing suppression function of P22.

### CsRPS21 Inhibits CCYV Accumulation in *N. benthamiana* Protoplasts

**FIGURE 5:**
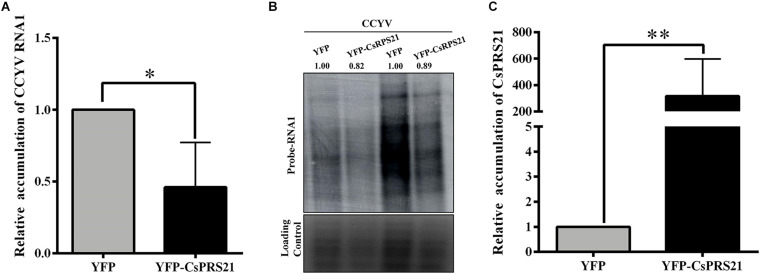
Transient expression of CsRPS21 in *N. benthamiana* protoplasts inhibited CCYV accumulation. **(A,B)** Accumulation of CCYV RNA1 of transfected protoplasts was measured using RT-qPCR and northern blotting with the probe of CCYV RNA1 at 24 hpi. **(C)** Protoplasts isolated from YFP-CsRPS21 and YFP agroinfiltrated *N. benthamiana* leaves were transfected with CCYV RNA1 and RNA2. Accumulation of CsRPS21 was measured using RT-qPCR at 24 hpi. Data were pooled across experiments and statistically analyzed using *t*-tests. Bars represents the mean ± standard deviation (SD). Single and double asterisks indicate significant differences at ^∗^*p* < 0.05 and ^∗∗^*p* < 0.01, respectively.

To investigate the role of CsRPS21 in CCYV replication, protoplasts were isolated from *N*. *benthamiana* leaves agroinfiltrated with YFP-CsRPS21 and transfected *in vitro* with CCYV RNA1 and RNA2 transcripts. At 1 dpi, the total RNA of the transfected protoplasts was extracted and analyzed by northern blotting and RT-qPCR. The results showed that, with the increased CsRPS21 expression, CCYV RNA1 accumulation was significantly decreased in YFP-CsRPS21-treated protoplasts compared with the YFP control (Figure 5), indicating the negative regulation of CCYV accumulation by CsRPS21.

## Discussion

RPs are involved in processes related to the cell cycle, apoptosis, development, oncogenesis, and control of rDNA transcription, in addition to their RNA chaperone activity (Jeon et al., 2008; Lindstrom, 2009; Kim et al., 2014). In *Arabidopsis thaliana*, knockout of plastid RP S21 impaired the photosynthesis activity and sugar response, possibly via reduced protein synthesis (Morita-Yamamuro et al., 2004). Besides, plastid RPS21 plays role in the response to C/N balance (Dong et al., 2020). In microorganisms, S21 is involved in stress resistance, growth, and motility (Akanuma et al., 2012; Metselaar et al., 2015; Koomen et al., 2018). Different RPs like RPL10, RPL13, RPL18, RPL24, and RPS6 are involved in viral infections of plants (Bureau et al., 2004; Rocha et al., 2008; Hafren et al., 2013; Zorzatto et al., 2015; Chen et al., 2016; Ivanov et al., 2016; Rajamaki et al., 2017; Li et al., 2018). The function of RP S21 during viral infections of plants is unknown. In our study we found that CsRPS21 interacted with CCYV P22 and negatively regulated CCYV P22 accumulaiton and silencing suppressor activity, and early viral accumulaiton. CCYV P22, a weak silencing suppressor, is able to suppress local RNA silencing induced by dsRNA (Orfanidou et al., 2019). Using *in vitro* RNA binding analysis P22 is able to bind to ss and ds long RNAs, in addition to ss and ds small interfering (si) RNA molecules *in vitro* (Salavert et al., 2020). A previous study reported that CCYV P22 interacts with SKP1 and F-box like domain of CCYV P22 is essential for the RNA silencing activity (Chen et al., 2019). In this study we found that the interaction down-regulated the CCYV P22 accumulation, and interfered with the silencing suppressor activity of CCYV P22 suggesting that various pathways are possibly involved in the process of CCYV P22 silencing suppression and host defense response.

ToCV P22 was found to be dispensable for viral replication (Landeo-Rios et al., 2016), so the effect on replication is likely due to interaction with viral proteins other than P22. Here, we used protoplasts to uncover the possible role of CsRPS21 in the early infection of CCYV (Figure 5). In a viral RNA transfection experiment, the earliest steps in virus life cycles, such as genomic RNA replication and translation, can be monitored because the virus propagation is restricted within single protoplasts (Zhu et al., 2014). Using northern blotting to detect CCYV RNA accumulaiton we found that overexpression of RPS21 in *N. benthamiana* protoplasts caused slight down-regulation of rRNAs indicating that RPS21 was negatively involved in regulating rDNA transcription. As previously reported RPS6 was involved in down-regulation of rRNA in *Arabidospsis* protoplast (Kim et al., 2014).

CsRPS21 shares a high identity with chloroplast RPS21 from cucurbits, although we found that cucumber RPS21 localized in both the nuclei and chloroplasts of *N*. *benthamiana* epidermal cells. The localization of other cucurbit RPS21s in *N*. *benthamiana* is unknown. In *Arabidopsis thaliana*, cytosolic RPS21 is localized in the cytosol and nuclear, and plastid RPS21 is localized in the chloroplast (Wang et al., 2018; Dong et al., 2020).

Analysis combining the Nuclear Localization and SMART Protein prediction tools indicated that the domains for interaction and nuclear localization overlapped (Figure 3), suggesting the importance of nuclear localization during the interaction. The full length of the domain is important for nuclear localization since both deletion mutants with half coverage of the domain were localized in both the cytoplasm and nuclei.

According to our result CsRPS21 interacts with three other CCYV encoded proteins besides P22 (Figure 2). It is possible that CsPRS21 plays multifunctional roles during viral infection through regulating different viral proteins at various infection stages. Currently, the function of P4.9, P2-6, and P59 was limitedly studied, the interaction will give a hint on the further study of P4.9, P2-6, and P59.

We identified a cucumber ribosomal-like protein, termed CsRPS21, that interacts with CCYV P22. A conserved CsRPS21 domain was identified and found to be indispensable for its nuclear localization and interaction with P22. Transient expression of CsRPS21 in *N. benthamiana* leaves interfered with the P22 accumulation to inhibit P22 silencing suppressor activity. In addition, CsRPS21 expression in *N*. *benthamiana* protoplasts inhibited CCYV accumulaiton. These results suggest that CsRPS21 negatively regulates CCYV infection, possibly by inhibition of P22 accumulation and silencing suppressor activity, and increase our knowledge of the function of RPs during viral infection. RPS21 probably represents one of the translation machinery that is usurped by CCYV P22 protein. It would be interesting to see if other RPs are involved in the recognition of P22.

## Data Availability Statement

The datasets presented in this study can be found in online repositories. The names of the repository/repositories and accession number(s) can be found in the article/Supplementary Material.

## Author Contributions

YS designed the experiment. YW, YJS, XH, SC, and LY performed the experiments. HL critically reviewed the manuscript. BS contributed to the data discussion. YS, XY, and YW wrote the manuscript. All authors read and approved the manuscript.

## Conflict of Interest

The authors declare that the research was conducted in the absence of any commercial or financial relationships that could be construed as a potential conflict of interest.

## Footnotes

1 http://nls-mapper.iab.keio.ac.jp/

## Supplementary Material

Supplementary Figure 1 Alignment of amino acid sequences of CsRPS21 from different cucurbit plants.

Supplementary Figure 2 YFP fluorescence was not detected in the combination of P22-nYFP + cYFP and nYFP + CsRPS21-cYFP. Bar scale represents 20 μm.

Supplementary Figure 3 **(A)** Colocalization of YFP-CsRPS21 and P22-RFP in *N. benthamiana*. Confocal images were taken at 2 dpi. Bar represents 20 μm. **(B)** Free YFP was expressed as a control. Bar scale represents 20 μm.

Supplementary Figure 4 YFP fluorescence was not detected in the combination of P22-nYFP + CsRPS21_1–9__0_-cYFP, P22-nYFP + cYFP, nYFP + CsRPS21_1–9__0_-cYFP, nYFP + CsRPS21_1–1__45_-cYFP. Bar scale represents 20 μm.

Supplementary Figure 5 GFP fluorescence intensity of Figure 4D was measured using ImageJ2 software. Thirty independent images for each group were measured and values were statistically analyzed with *t*-tests. Three biological repeats were performed.

Supplementary Table 1 Primers used in the paper.
