# Supplementary material for: Cucumber Ribosomal Protein CsRPS21 Interacts With P22 Protein of Cucurbit Chlorotic Yellows Virus
Source: Front Microbiol. 2021 Apr 29;12:654697. doi: 10.3389/fmicb.2021.654697 (PMC8116660; doi:10.3389/fmicb.2021.654697)
Supplement: Supplementary Figure 1 — Alignment of amino acid sequences of CsRPS21 from different cucurbit plants. [file Data_Sheet_1.pdf]

Cucumber ribosomal protein CsRPS21 interacts with P22 protein of cucurbit chlorotic yellows virus

Xue Yang[1, †], Ying Wei[1, †], Yajuan Shi [1, †], Xiaoyu Han[1], Siyu Chen[1], Lingling Yang[1], Honglian Li[1], Bingjian Sun[1], Yan Shi[1, *]

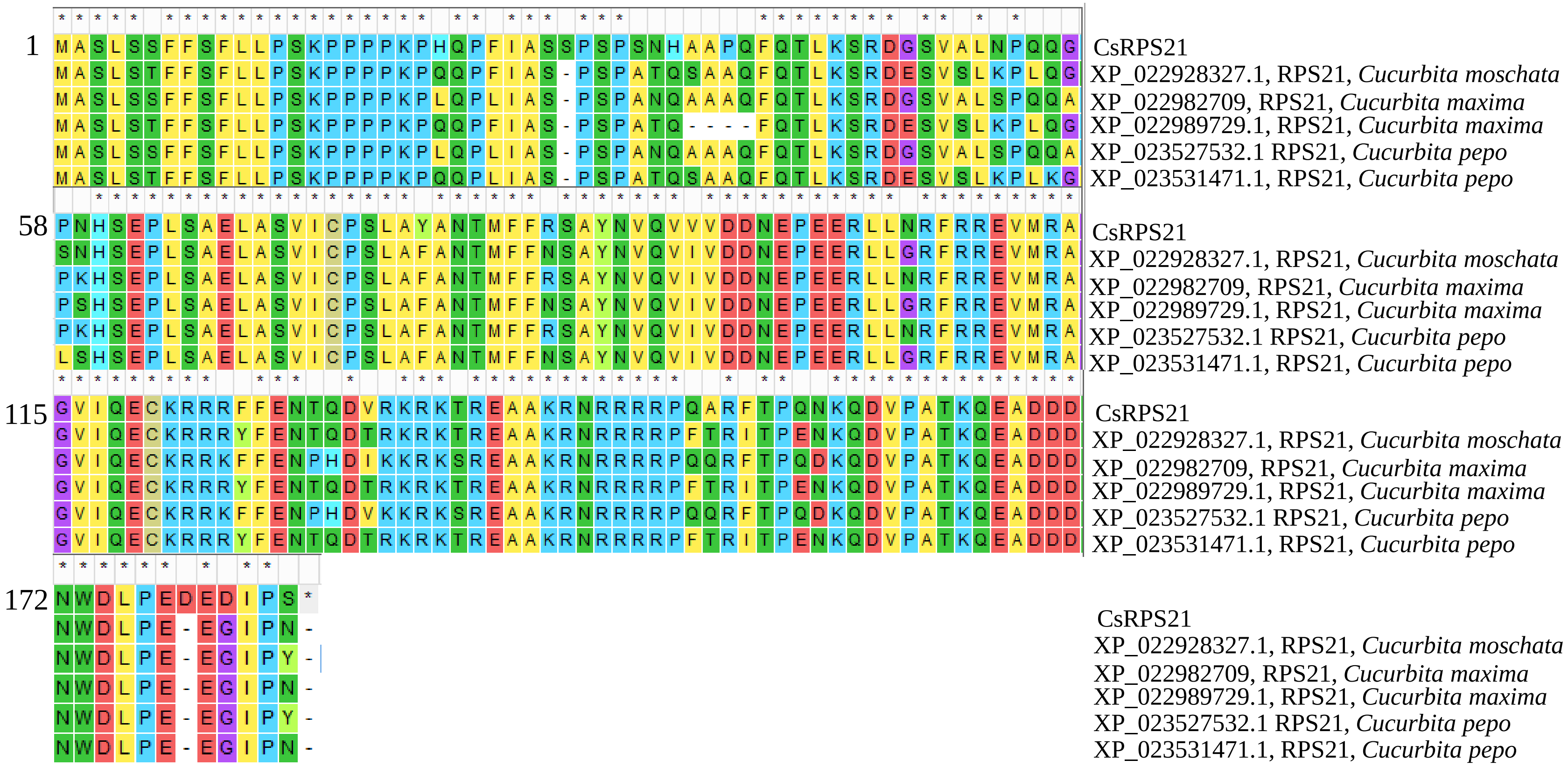

Supplementary Figure S1. Alignment of amino acid sequences of RPS21 from different cucurbit plants

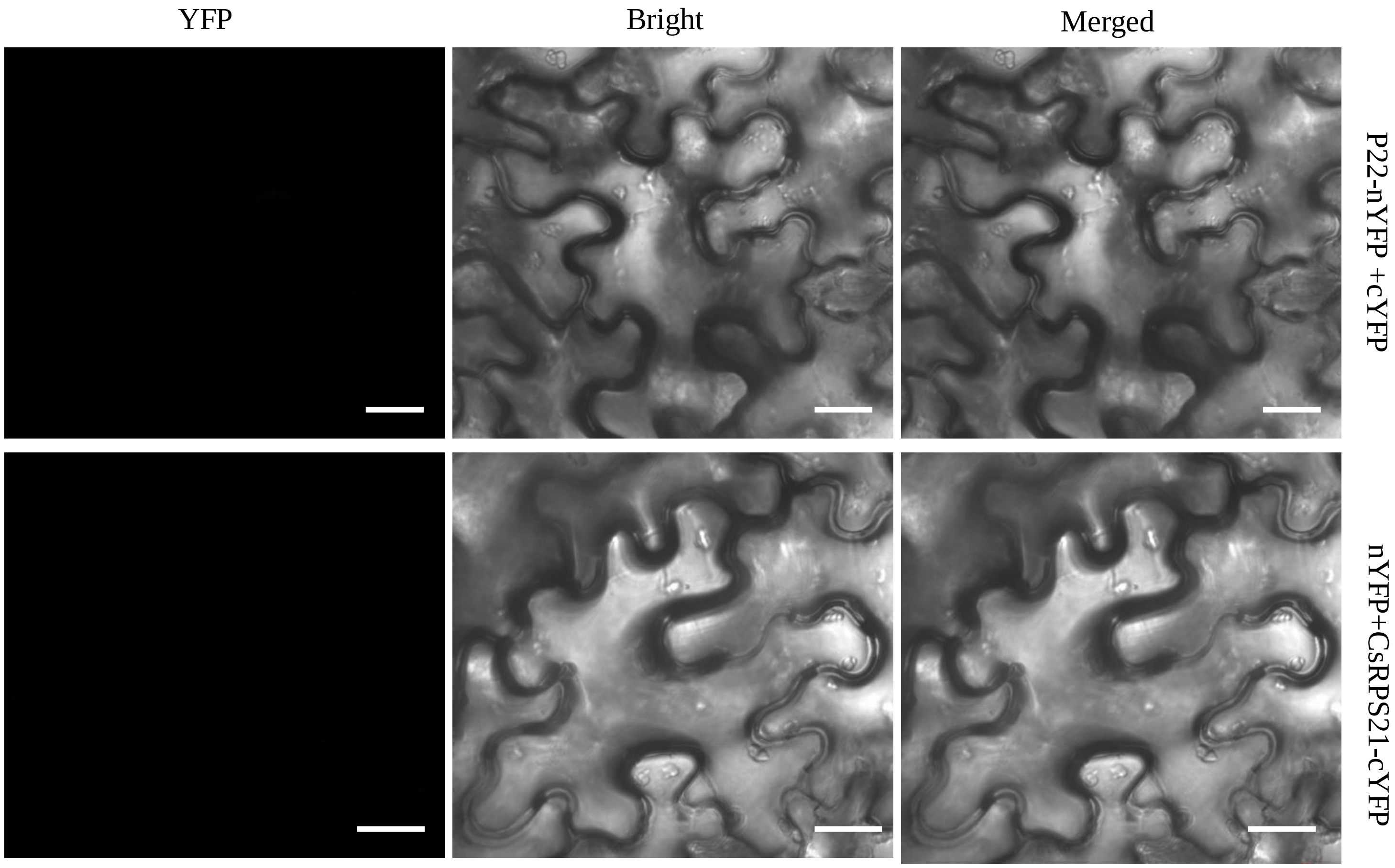

Supplementary Figure S2. YFP fluorescence was not detected in the combination of P22-nYFP +cYFP and nYFP+CsRPS21-cYFP. Bar scale represents 20 μm.

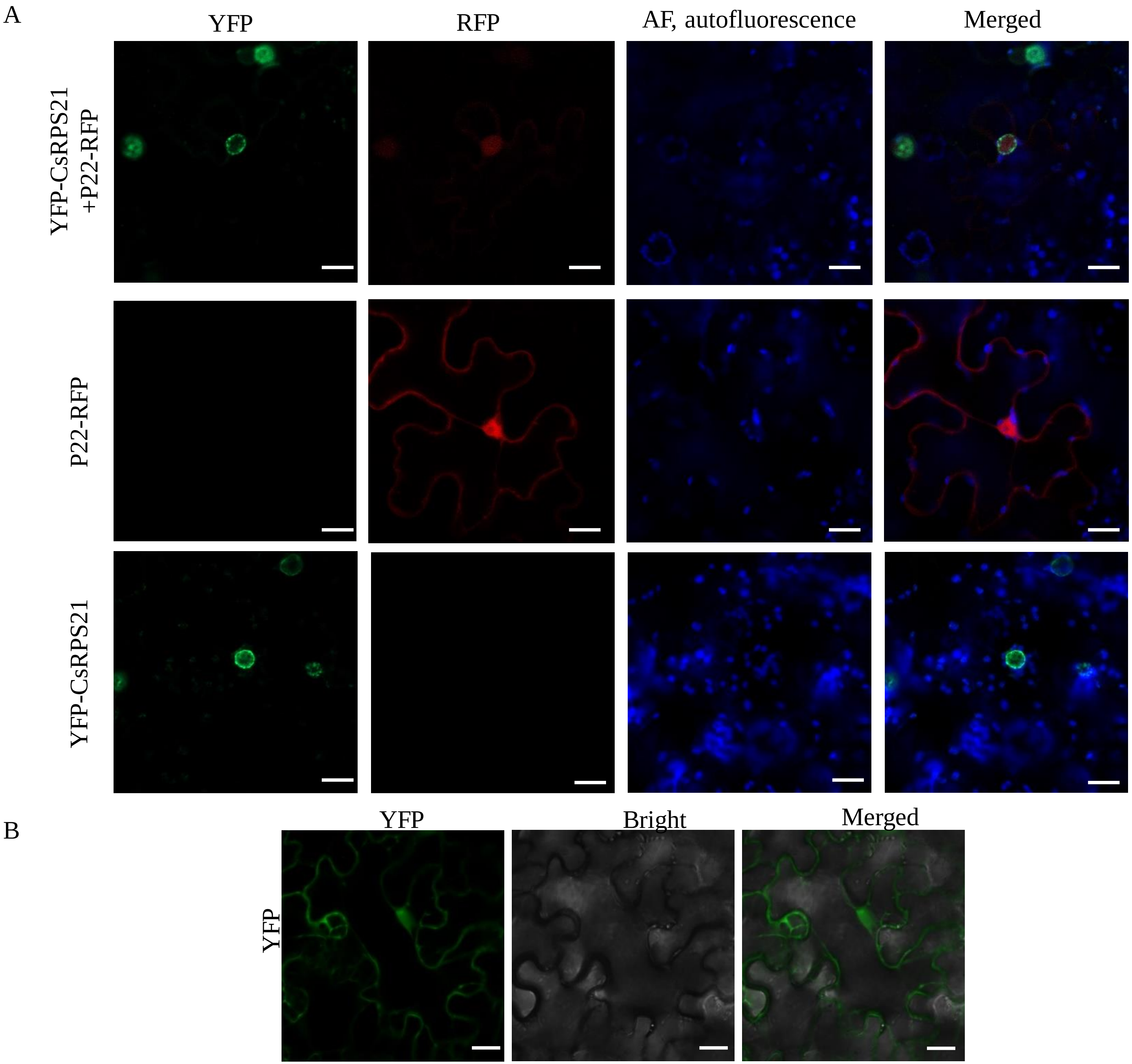

Supplementary Figure S3. (A) Colocalization of YFP-CsRPS21 and CFP-P22 was in the nuclear. Bar scale represents 20 µm. (B) YFP only expressed as a control in sub cellular localization experiments. Bar scale represents 20 µm.

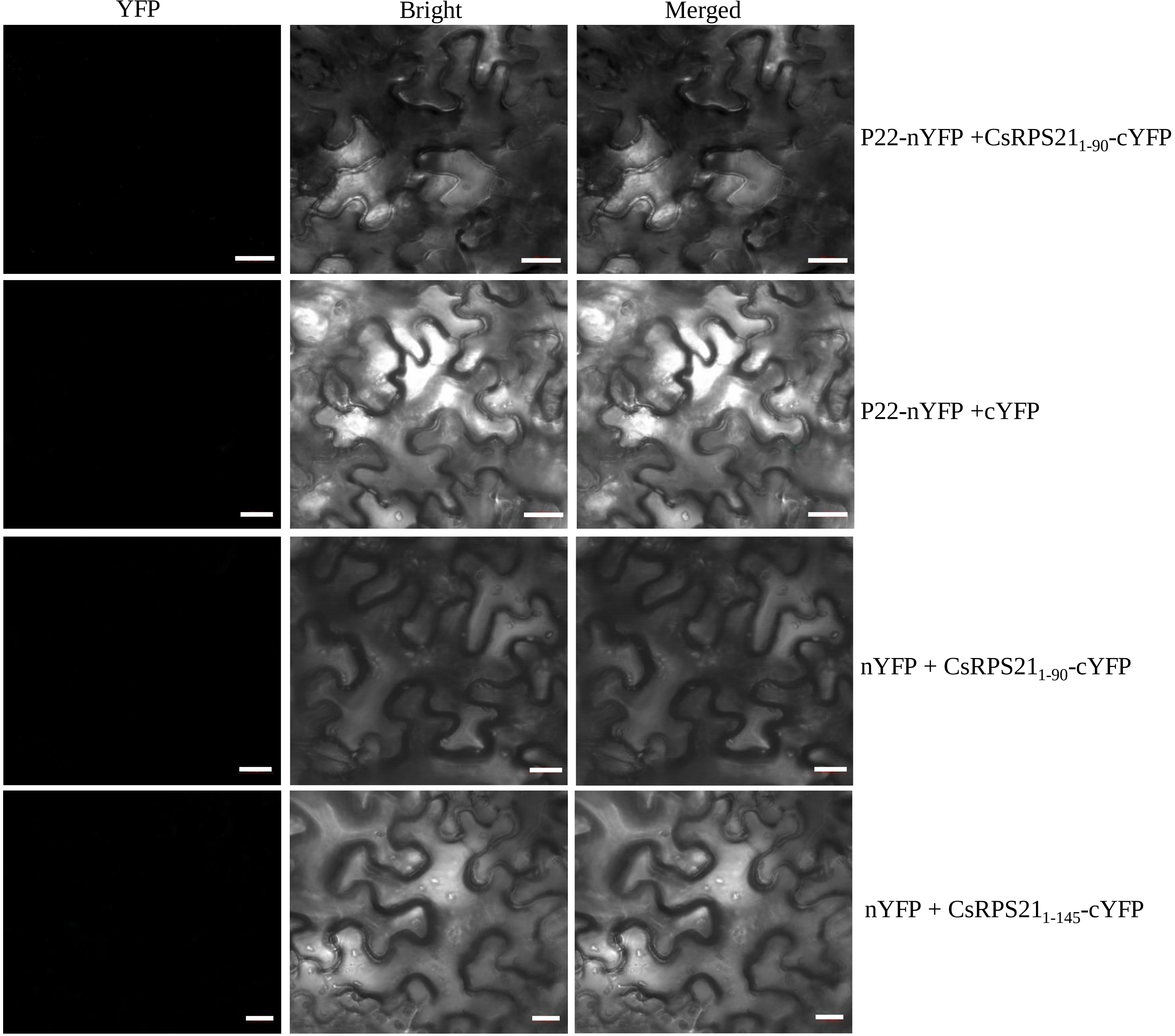

Supplementary Figure S4. YFP fluorescence was not detected in the combination of P22-nYFP +CsRPS21$_{1\text{-}90}$-cYFP , P22-nYFP +cYFP, nYFP+CsRPS21$_{1\text{-}90}$-cYFP, nYFP+CsRPS21$_{1\text{-}145}$-cYFP. Bar scale represents 20 μm.

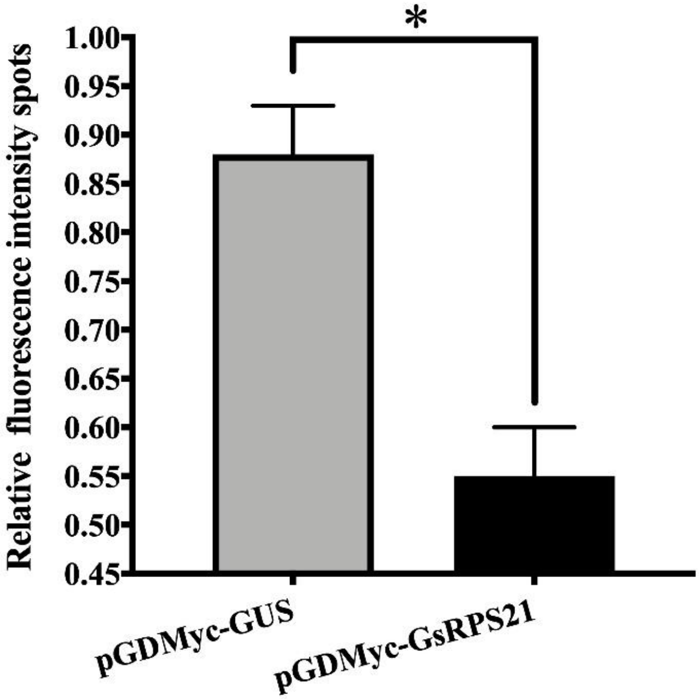

Supplementary Figure S5. GFP fluorescence intensity of Fig. 4D was measured using ImageJ2 software. Thirty independent images for each group were measured and values were statistically analyzed with t-tests. Three biological repeats were performed.

Table S1 Primers used in the paper

Primers	Sequences (5'-3')
BDP22F	GGAATTCCATATGATGAATAATCGTAAATTTTTC
BDP22R	CCTGGATCCTTATATTACGAACTTATTAAG
ADCsRS21F	ACCGAATTCATGGCTTCCCTCTCCAGCTTC
ADCsRPS21R	GTACTCGAGTTAACTAGGAATGTCCTCATC
ADCsRPS21$_{145}$R	GTACTCGAGTTAGCGGTTGCGTTTAGCAGC
ADCsRPS21$_{128}$F	ACCGAATTCAACACTCAAGATGTTAGGAAGCG
ADCsRPS21$_{127}$R	GTACTCGAGTTA CTCAAAGAATCTCCGTCTCTTA
ADCsRPS21$_{91}$F	ACCGAATTC CAGGTGGTTGTGGATGATAATGA
ADCsRPS21$_{90}$R	GTACTCGAGTTAAACATTATATGCGGATCTGAAGAAC
BPP22F	GGGGACAAGTTTGTACAAAAAAGCAGGCTTCATGAATAATCGTAAATTTTTCG
BPP22R	GGGGACCACTTTGTACAAGAAAGCTGGGTCTATTACGAACTTATTAGAG
BPCsRPS21F	GGGGACAAGTTTGTACAAAAAAGCAGGCTTCATGGCTTCCCTCTCCAGC
BPCsRPS21R	GGGGACCACTTTGTACAAGAAAGCTGGGTCACTAGGAATGTCCTCATC
BPCsRPS21$_{91}$F	GGGGACAAGTTTGTACAAAAAAGCAGGCTTCATGCAGGTGGTTGTGGATGATAATGA
BPCsRPS21$_{145}$R	GGGGACCACTTTGTACAAGAAAGCTGGGTCGCGGTTGCGTTTAGCAGC
BPCsRPS21$_{127}$R	GGGGACCACTTTGTACAAGAAAGCTGGGTCCTCAAAGAATCTCCGTCTCTTA
BPCsRPS21$_{128}$F	GGGGACAAGTTTGTACAAAAAAGCAGGCTTCATGAACACTCAAGATGTTAGGAAGCG
FLAGP22F	CGCGTCGACGATGAATAATCGTAAATTTTTC
FLAGP22R	GGTGGATCCTTATATTACGAACTTAT
MycRPS21F	TATCTGCAGATGGCTTCCCTCTCCAGCT
MycRPS21R	AACGGATCCTTAACTAGGAATGTCCTCA
QRT-Nb-ActinF	TTGTTAGGGATGTGAAGGA
QRT-Nb-ActinR	CATGATGGAATTGTATGTGG
QRT-RPS21F	TCAACAGGGTCCCAACCATT
QRT-RPS21R	TAACCTCTCGCCGGAATCTG

QRT-RNA1F	TAAACTAAAGCAGCAAGGTG
QRT-RNA1R	GTGGTACTCTATTGATGAAAGG
Probe-CCYV RNA1F	TAATACGACTCACTATAGGGCTGTGCCTCGACCTTATC
Probe-CCYV RNA1R	TGGACCAATGACAATCTC
Probe-GFPF	TAATACGACTCACTATAGGGATGGTGAGCAAGGGCGAG
Probe-GFPR	TCAAAGATCTACCATGTA
